# Cross-border travel patterns affect magnitude estimates for the Ebola Bundibugyo epidemic

**DOI:** 10.1038/s44360-026-00178-y

**Published:** 2026-07-17

**Authors:** Younjung Kim, Cathal Mills, Christl A. Donnelly

**Affiliations:** 1 Department of Statistics, University of Oxford, Oxford, UK; 2 Pandemic Sciences Institute, University of Oxford, Oxford, UK

**Keywords:** Infectious diseases, Health policy

## Abstract

A geographic spread approach is often used to estimate the true burden of infectious disease cases in a source population by leveraging statistical signals generated by cases exported and reported elsewhere. Here we estimated an upper bound on the likely burden of Bundibugyo virus disease cases in the Democratic Republic of Congo early in the unfolding 2026 epidemic based on the number of cases imported and confirmed in Uganda. We expanded a geographic spread approach to account for its specific epidemiological contexts: most cross-border movements between the Democratic Republic of Congo and Uganda are short-duration trips, and, at the time of this analysis, all cases imported and confirmed in Uganda were individuals who travelled specifically to seek healthcare. Incorporating these factors substantially affected burden estimates, highlighting both the potential severity of the epidemic in its early phase and the critical need to consider local epidemiological contexts when applying geographic spread approaches to reduce and be aware of potential biases in estimates for early epidemic responses.

## Abstract (web summary)

Incorporating local information on trip frequency, duration, and purpose, including healthcare-seeking travel behaviour, indicates that the magnitude of the recent Ebola Bundibugyo epidemic in the DRC may have been substantially underestimated previously.

## Main

Soon after the detection of an epidemic (when epidemiological data are limited), early epidemic responses can be informed by estimating the total case burden in the affected region, leveraging statistical signals generated by cases exported elsewhere^1,2^. This ‘geographic spread’ approach must carefully consider the specific epidemiological contexts that generate these signals. Notably, when travel durations are short relative to the infection-to-detection interval, infected travellers may return home before detection, unlike prior applications of this approach that leveraged international air travel where most passengers are likely to remain abroad long enough to be detected as cases there^1,2^. In addition, in settings where short-term cross-border travel is relatively easy via alternative means (for example, by foot, car or train), infected individuals would be more likely to attempt travelling elsewhere to seek healthcare.

These two contexts directly apply to the 2026 Bundibugyo virus disease (BVD) epidemic in the Democratic Republic of Congo (DRC), where the affected regions share a long and highly porous border with Uganda. When applying a geographic spread approach to this epidemic, it is therefore critical (1) to account for the duration and frequency of international travel from the DRC and (2) to ensure that the epidemiological histories of exported cases are consistent with the modelling assumption that travel is unrelated to infection status.

First, until 21 June 2026, all 15 BVD cases imported and confirmed in Uganda were “Congolese nationals who came to Uganda to seek medical care”^3,4^. For example, the first two confirmed imported cases were recently described as “self-referrals from the epicentre in Ituri province seeking advanced care in Uganda”^5^, and the third confirmed imported case sought “care at a private hospital in the capital Kampala”^6^. This healthcare-seeking behaviour violates the key assumption of the conventional geographic spread approach that individuals in the source population have an equal probability of travelling regardless of infection status^1,2^. The reality is that, excluding travel for healthcare, there had been no confirmed imported cases in Uganda up to 21 June 2026^3,4^.

Second, according to the March 2020 Uganda–DRC Border Flow Monitoring Dashboard by the International Organization for Migration (IOM), 90.6% of trips to Uganda from Ituri and Nord Kivu, the DRC, lasted a week or less, with 64.7% comprising day trips^7^. Similar trends were observed in the April 2019 Uganda–DRC Border Flow Monitoring Dashboard data, with 92.8% of trips lasting a week or less^8^. While trip-duration data were not available for the period when early imported cases were confirmed in Uganda, short-duration trips are considered to “have dominated the cross-border movements along the DRC–Uganda border during this period” (H. Kyobe Bosa, personal communication) with communities along the border operating as dynamic and shared socioeconomic entities. This unique border context underscores the possibility that infected individuals may remain undetected while travelling, as demonstrated by a case having been confirmed only after returning to the DRC^6^, clearly distinguishing the current epidemic from those that were previously analysed using the geographic spread approach assuming that international air travel passengers remained abroad long enough to be detected^1,2^.

**Fig. 1:**
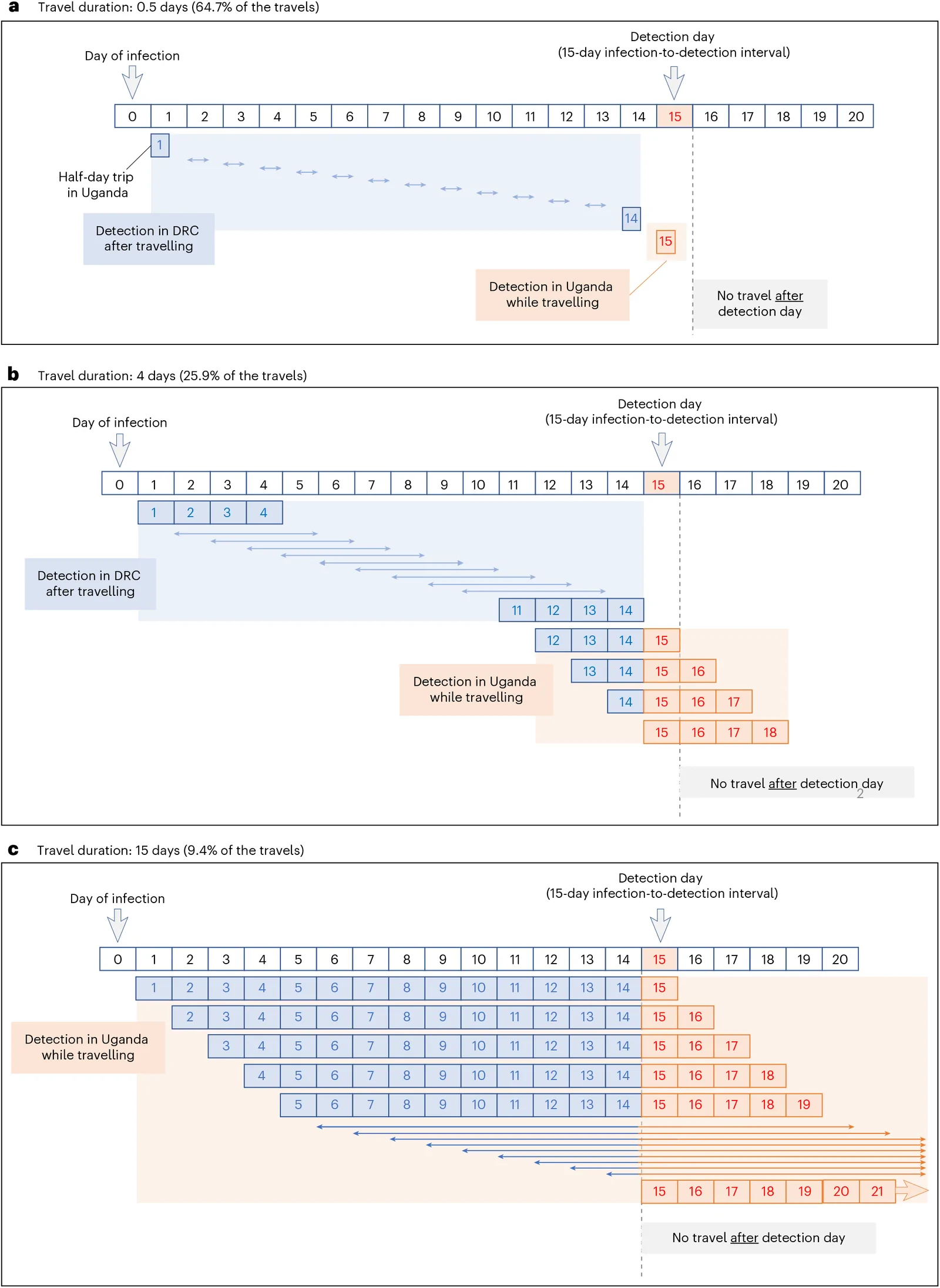
Schematic description of BVD case detection scenarios in Uganda by travel duration. a–c, BVD case detection scenarios with trip durations of 0.5 (**a**), 4 (**b**) and 15 (**c**) days, respectively. The numbers in the rectangular boxes represent days since infection.The percentage for each travel duration category was obtained by normalizing the data reported in the March 2020 Uganda–DRC Border Flow Monitoring Dashboard^7^ to exclude responses with unknown travel durations.

Figure 1 illustrates these scenarios across different travel durations, assuming a fixed 15-day infection-to-detection interval (in the actual analysis, we obtained the overall probability of detection while travelling as a weighted average over different durations of travel and different infection-to-detection intervals; Methods). Infected individuals travelling to Uganda ‘for less than a day’ can only be detected in Uganda if they travel just before detection (that is, on the day of detection) (Fig. 1a). Similarly, those travelling to Uganda ‘for 4 days’ will not be detected in Uganda as long as they return to the DRC before day 15 (Fig. 1b). By contrast, infected individuals travelling to Uganda for a duration ‘equal to or longer than 15 days’ will be detected in Uganda regardless of when they begin their travel relative to the time of infection (Fig. 1c).

Accounting for different scenarios of trip durations and excluding healthcare-seeking travellers substantially impact the estimates for the BVD case burden in the DRC (see Table 1 for results by the scenarios explored and the Methods for methodological details). With three imported cases, accounting for the observed pattern of trip durations from Ituri and Nord Kivu, the DRC, to Uganda^7,8^ substantially increased the central estimates and widened the 95% confidence intervals compared to not accounting for travel durations within our framework and to those reported recently^9,10^. With zero imported cases (that is, after excluding the three imported cases given their healthcare-seeking travel behaviour) and accounting for trip durations, the central estimates and the lower confidence limits were uninformative (zero by definition); however, the upper confidence limits provided informative likely upper bounds for the early BVD burden in the DRC, which can be used to inform epidemic responses; if the true BVD cases in the DRC were higher than these upper limits, non-zero imported cases would have been expected among individuals travelling to Uganda for general purposes, including healthcare not specifically related to Bundibugyo virus infection (visits for healthcare represented less than 1% in the March 2020 Uganda–DRC Border Flow Monitoring Dashboard data^7^).

**Table 1:** Estimates (and 95% confidence intervals, CIs) of the number of BVD cases in the DRC, assuming three or zero imported cases from Ituri and Nord Kivu, the DRC, to Uganda, with and without accounting for travel duration in Uganda

Source population and travellers considered	The daily number of travellers to Uganda from the source population^a,b^	Source population size^a^	Trip duration scenario^c^	Estimated number of BVD cases in DRC (95% CI)		
				Median infection-to-detection interval of 10 days	Median infection-to-detection interval of 15 days	Median infection-to-detection interval of 20 days
Assuming three imported cases in Uganda (note: upon detailed investigation, it was found that these imported cases travelled to Uganda to seek healthcare^5,6^)						
Ituri only						
All travellers	1,871	4,392,200	Baseline trip durations	3,024 (752–7,841)	2,515 (626–6,523)	2,154 (536–5,584)
			Assuming all trips were long	683 (170–1,771)	460 (114–1,193)	347 (86–899)
Non-day-travellers	660		Baseline trip durations	3,511 (873–9,105)	2,844 (707–7,375)	2,390 (594–6,197)
Ituri + Nord Kivu						
All travellers	4,339	13,392,200	Baseline trip durations	3,975 (989–10,309)	3,307 (822–8,576)	2,831 (704–7,342)
			Assuming all trips were long	898 (223–2,329)	605 (150–1,568)	456 (113–1,182)
Non-day-travellers	1,531		Baseline trip durations	4,617 (1,148–11,971)	3,739 (930–9,697)	3,142 (781–8,148)
Assuming zero imported cases in Uganda, excluding imported cases who travelled for healthcare						
Ituri only						
All travellers	1,871	4,392,200	Baseline trip durations	0 (0–1,936)	0 (0–1,610)	0 (0–1,379)
			Alternative trip durations	0 (0–1,613)	0 (0–1,428)	0 (0–1,282)
			Assuming all trips were long	0 (0–437)	0 (0–294)	0 (0–222)
Non-day-travellers	660		Baseline trip durations	0 (0–2,248)	0 (0–1,821)	0 (0–1,530)
Ituri + Nord Kivu						
All travellers	4,339	13,392,200	Baseline trip durations	0 (0–2,545)	0 (0–2,117)	0 (0–1,813)
			Alternative trip durations	0 (0–2,121)	0 (0–1,878)	0 (0–1,685)
			Assuming all trips were long	0 (0–575)	0 (0–387)	0 (0–292)
Non-day-travellers	1,531		Baseline trip durations	0 (0–2,956)	0 (0–2,394)	0 (0–2,012)

^a^The numbers of daily travellers and the source population sizes were as published by McCabe et al.^9^.

^b^The numbers of non-day-travellers were obtained by multiplying the proportion of travellers visiting Uganda longer than 1 day among those who reported travel duration in the March 2020 Uganda–DRC Border Flow Monitoring Dashboard^7^.

^c^Baseline trip duration: ‘less than 1 day’ = 64.7%, ‘1 day to 1 week’ = 25.9%, ‘longer than 1 week’ = 9.4% (following the March 2020 report^7^); alternative trip duration: ‘less than 1 day’ = 47.4%, ‘1 day to 1 week’ = 45.4%, ‘longer than 1 week’ = 7.2% (following the April 2019 report^8^).

We note that our estimates are expected to change proportionally to the assumed source population size and could thus be biased if the assumed source population sizes did not accurately reflect the catchment population of those travelling to Uganda. For example, those residing near the DRC–Uganda border probably have a substantially different probability of travelling to Uganda compared with those living further away from the border. This is particularly relevant considering the operation of border communities as dynamic and shared economic entities^7,8^. Furthermore, individuals who can afford to travel to seek healthcare abroad may also have different baseline infection risks compared with the general population. This knowledge gap suggests that a better understanding of the complex cross-border crossing dynamics will help obtain more robust estimates of the true case burden using this geographic spread approach. This necessity, in turn, underscores the importance of incorporating epidemiological perspectives into the design of border surveillance activities (for example, flow monitoring by the IOM^7,8^) in regions where the risk of high-consequence infectious disease outbreaks is considered high.

Our analysis demonstrates the importance of allowing for key epidemiological contexts when using a geographic spread approach to avoid potentially biasing estimates that are critical to inform early epidemic responses. While the method is appealingly simple and intuitive, its reliability and generalizability depend on a careful consideration of these real-world contexts.

## Methods

### Travel duration

The geographic spread approach was previously applied to estimate H1N1 pandemic influenza cases in Mexico in 2009^1^ and early COVID-19 cases in Wuhan in 2019^2^, as well as McCabe et al.^9,10^ for the current BVD epidemic. However, the baseline assumptions of these applications were that infected or symptomatic individuals would be detected in their destinations during travelling regardless of their travel duration, which may not apply to the DRC–Uganda border context. The methodological generalization of the geographic spread approach to account for travel duration was derived recently to estimate the burden of clinically significant hantavirus cases in Argentina based on a case detected on a cruise ship, leveraging the average duration of cruise trips^11^.

When travel durations are short relative to the interval from infection to detection, it is possible that infected travellers return to the DRC (the epidemic source region) and therefore are not detected in Uganda. In reality, the time from infection-to-detection varies between individuals depending on biological and behaviour factors as well as the availability of healthcare for clinical assessment and diagnostic testing. Therefore, we generalized the method further to obtain the overall probability of detection while travelling as a weighted average over different durations of travel and different infection-to-detection intervals (see ‘Estimation of true burden of BVD cases in the DRC’ section).

### Zero exported cases from DRC to Uganda

Until 21 June 2026, all 15 BVD cases imported and confirmed in Uganda were “Congolese nationals who came to Uganda to seek medical care”^3,4^. In particular, the first two confirmed imported cases were recently described as “self-referrals from the epicentre in Ituri province seeking advanced care in Uganda”^5^, and the third confirmed imported case sought “care at a private hospital in the capital Kampala”^6^.

This healthcare-seeking travel behaviour violates the key assumption of the usual geographic spread approach that individuals in the source population have an equal probability of travelling regardless of infection status and indicates that, excluding travel for healthcare, there had been no exported cases from DRC to Uganda by 9 June 2026, when McCabe et al.^10^ estimates were published based on the first three confirmed imported cases and up until 21 June 2026, as of this writing.

### Data

#### Travel duration from Ituri and Nord Kivu to Uganda

For the baseline scenario, data on the distribution of travel durations from Ituri and Nord Kivu to Uganda were obtained from the March 2020 Uganda–DRC Border Flow Monitoring Dashboard (Baseline scenario)^7^. We reclassified travel duration categories into three categories and calculated the proportion of each category by normalizing the reported data to exclude unknown travel durations: (a) less than 1 day (64.7%), (b) 1 day to 1 week (25.9%) and (c) longer than 1 week (9.4%). For the alternative scenario, data were obtained from the April 2019 Uganda–DRC Border Flow Monitoring Dashboard (Alternative scenario)^8^: (a) less than 1 day (47.4%), (b) 1 day to 1 week (45.4%) and (c) longer than 1 week (7.2%).

#### Daily number of travellers from Ituri and Nord Kivu to Uganda and source population size

Data on the daily number of travellers from Ituri (*n* = 1,871) and both Ituri and Nord Kivu (*n* = 4,339) to Uganda were obtained from McCabe et al.^9^. Using the same data allowed the direct assessment on the impact of accounting travel duration and the number of exported cases analysed. For the analyses focusing on non-day travellers, we scaled the daily number of travellers by the proportion of non-day travellers (35.3%) obtained from the March 2020 Uganda–DRC Border Flow Monitoring Dashboard March 2020^7^: Ituri (*n* = 660) and both Ituri and Nord Kivu (*n* = 1,531). The corresponding source population sizes were also obtained from McCabe et al.^9^: Ituri (*n* = 4,392,200) and both Ituri and Nord Kivu (*n* = 13,392,200).

#### Estimation of true burden of BVD cases in the DRC

For a given source population (Ituri, or Ituri and Nord Kivu), the daily probability of travelling to Uganda, ${P}_{\mathrm{travel}}$, was calculated by dividing the daily number of travellers by the source population size^9^. The delay from infection to detection was modelled using a Gamma distribution for the incubation period^10^ (shape 2.52, rate 0.40), shifted by an assumed delay from symptom onset to clinical detection to give median times from infection to detection of 10, 15 and 20 days, following McCabe et al.^9^, and discretized at the day level.

For each category of travel duration *i*, the probability that an infected individual was detected in Uganda while travelling, ${P}_{\mathrm{detection},{i}}$, was calculated over a grid of possible travel days and infection-to-detection intervals. For the ‘less than 1 day’ and ‘1 day to 1 week’ travel duration categories, we assigned the fixed median travel durations of 0.5 and 4 days, respectively. For the ‘longer than 1 week’ category, we assumed an indefinite stay, indicating that infected travellers remained in Uganda long enough to be detected there. Finally, we obtained the overall probability that an infected individual was detected in Uganda while travelling, ${P}_{\mathrm{detection},\mathrm{overall}}$, by weighting each duration-specific probability, ${P}_{\mathrm{detection},i}$, by the proportion of each travel duration category. Individuals were assumed not to travel between detection and recovery.

Finally, the number of exported cases was divided by the overall probability of detection to estimate the cases in the DRC, with the 95% confidence interval being obtained from dividing the confidence limits for the rate of exported cases by the overall probability of detection. For example, the 95% Poisson-likelihood-based confidence interval for the rate of exported cases based on observing zero exported cases is (0, 1.92).

If infected individuals were less likely to travel for non-healthcare-seeking purposes, for example due to feeling poorly, the burden estimate would scale up (according to the inverse of the factor by which individuals infected with the Bundibugyo virus were less likely to travel).

### Reporting summary

Further information on research design is available in the Nature Portfolio Reporting Summary linked to this article.

## Supplementary information

Reporting Summary

## Data Availability

The study was conducted using publicly available data. All data sources are referenced and detailed in the Methods.

## Code availability

The underlying code is available as an R package, EpiSOURCE (Epidemiological Surveillance of OUtbreaks via Reported Case Exports), available via GitHub at https://github.com/KimYounjung/EpiSOURCE.
