# Supplementary material for: Cross-border travel patterns affect magnitude estimates for the Ebola Bundibugyo epidemic
Source: Nat Health. 2026 Jul 17;1(11):1200–4. doi: 10.1038/s44360-026-00178-y (PMC13727669; doi:10.1038/s44360-026-00178-y)
Supplement: Supplementary file 1 [file 44360_2026_178_MOESM1_ESM.pdf]

nature portfolio

Corresponding author(s): Christl A. Donnelly

Last updated by author(s): Jun 27, 2026

# Reporting Summary

Nature Portfolio wishes to improve the reproducibility of the work that we publish. This form provides structure for consistency and transparency in reporting. For further information on Nature Portfolio policies, see our Editorial Policies and the Editorial Policy Checklist.

## Statistics

For all statistical analyses, confirm that the following items are present in the figure legend, table legend, main text, or Methods section.

| n/a | Confirmed | |
|---|---|---|
| ☐ | ☒ | The exact sample size ($n$) for each experimental group/condition, given as a discrete number and unit of measurement |
| ☒ | ☐ | A statement on whether measurements were taken from distinct samples or whether the same sample was measured repeatedly |
| ☒ | ☐ | The statistical test(s) used AND whether they are one- or two-sided<br>*Only common tests should be described solely by name; describe more complex techniques in the Methods section.* |
| ☒ | ☐ | A description of all covariates tested |
| ☐ | ☒ | A description of any assumptions or corrections, such as tests of normality and adjustment for multiple comparisons |
| ☐ | ☒ | A full description of the statistical parameters including central tendency (e.g. means) or other basic estimates (e.g. regression coefficient) AND variation (e.g. standard deviation) or associated estimates of uncertainty (e.g. confidence intervals) |
| ☒ | ☐ | For null hypothesis testing, the test statistic (e.g. $F$, $t$, $r$) with confidence intervals, effect sizes, degrees of freedom and $P$ value noted<br>*Give P values as exact values whenever suitable.* |
| ☒ | ☐ | For Bayesian analysis, information on the choice of priors and Markov chain Monte Carlo settings |
| ☒ | ☐ | For hierarchical and complex designs, identification of the appropriate level for tests and full reporting of outcomes |
| ☒ | ☐ | Estimates of effect sizes (e.g. Cohen's $d$, Pearson's $r$), indicating how they were calculated |

*Our web collection on statistics for biologists contains articles on many of the points above.*

## Software and code

Policy information about availability of computer code

| | |
|---|---|
| Data collection | The study was conducted using publicly available data. All data sources are referenced and detailed in the Methods. |
| Data analysis | The underlying code is available as an R package, EpiSOURCE (Epidemiological Surveillance of OUtbreaks via Reported Case Exports), available at: https://github.com/KimYounjung/EpiSOURCE |

For manuscripts utilizing custom algorithms or software that are central to the research but not yet described in published literature, software must be made available to editors and reviewers. We strongly encourage code deposition in a community repository (e.g. GitHub). See the Nature Portfolio guidelines for submitting code & software for further information.

## Data

Policy information about availability of data

All manuscripts must include a data availability statement. This statement should provide the following information, where applicable:

- Accession codes, unique identifiers, or web links for publicly available datasets
- A description of any restrictions on data availability
- For clinical datasets or third party data, please ensure that the statement adheres to our policy

In the data availability statement we say: "The study was conducted using publicly available data. All data sources are referenced and detailed in the Methods."

## Research involving human participants, their data, or biological material

Policy information about studies with human participants or human data. See also policy information about sex, gender (identity/presentation), and sexual orientation and race, ethnicity and racism.

| | |
|---|---|
| Reporting on sex and gender | We do not report any data stratified by sex or gender. |
| Reporting on race, ethnicity, or other socially relevant groupings | We do not report any data stratified by race, ethnicity or other groupings. |
| Population characteristics | We used publicly available data on cross-border travel. |
| Recruitment | We did not recruit any participants. |
| Ethics oversight | No ethical approval was required as we did not collect data or recruit participants. |

Note that full information on the approval of the study protocol must also be provided in the manuscript.

# Field-specific reporting

Please select the one below that is the best fit for your research. If you are not sure, read the appropriate sections before making your selection.

☐ Life sciences ☒ Behavioural & social sciences ☐ Ecological, evolutionary & environmental sciences

For a reference copy of the document with all sections, see nature.com/documents/nr-reporting-summary-flat.pdf

# Behavioural & social sciences study design

All studies must disclose on these points even when the disclosure is negative.

| | |
|---|---|
| Study description | We used quantitative data on Bundibugyo virus disease (BVD) cases confirmed in Uganda, population sizes for Democratic Republic of Congo regions in which BVD is transmitting and data on cross-border travel (trip numbers and durations). All of these were publicly available. |
| Research sample | We did not sample any populations. |
| Sampling strategy | We did not sample any populations. |
| Data collection | All of the data were publicly available, identified through assessment of relevant published papers and online reports on migration from the International Organization for Migration. |
| Timing | We did not collect data. |
| Data exclusions | This is not applicable to this study used observed, but not sampled, data. |
| Non-participation | There were no study participants. This study used observed, but not sampled, data. |
| Randomization | There was no randomization. |

# Reporting for specific materials, systems and methods

We require information from authors about some types of materials, experimental systems and methods used in many studies. Here, indicate whether each material, system or method listed is relevant to your study. If you are not sure if a list item applies to your research, read the appropriate section before selecting a response.

## Materials & experimental systems

| n/a | Involved in the study |
| --- | --- |
| ☒ | ☐ Antibodies |
| ☒ | ☐ Eukaryotic cell lines |
| ☒ | ☐ Palaeontology and archaeology |
| ☒ | ☐ Animals and other organisms |
| ☒ | ☐ Clinical data |
| ☒ | ☐ Dual use research of concern |
| ☒ | ☐ Plants |

## Methods

| n/a | Involved in the study |
| --- | --- |
| ☒ | ☐ ChIP-seq |
| ☒ | ☐ Flow cytometry |
| ☒ | ☐ MRI-based neuroimaging |
